# A review of the clinical outcomes in idursulfase-treated and untreated Filipino patients with mucopolysaccharidosis type II: data from the local lysosomal storage disease registry

**DOI:** 10.1186/s13023-021-01875-5

**Published:** 2021-07-21

**Authors:** Marie Julianne C. Racoma, Maria Kristina Karizza B. Calibag, Cynthia P. Cordero, Mary Ann R. Abacan, Mary Anne D. Chiong

**Affiliations:** 1 Division of Clinical and Metabolic Genetics, Department of Pediatrics, Philippine General Hospital, University of the Philippines, Manila, Philippines; 2 Department of Rehabilitation Medicine, Philippine General Hospital, University of the Philippines, Manila, Philippines; 3 Department of Clinical Epidemiology, College of Medicine, University of the Philippines, Manila, Philippines; 4 Institute of Human Genetics, National Institutes of Health, University of the Philippines, Manila, Philippines; 5 Department of Biochemistry, Molecular Biology and Nutrition, University of Sto.Tomas - Faculty of Medicine & Surgery, Manila, Philippines

**Keywords:** Mucopolysaccharidosis type II, Hunter syndrome, Enzyme replacement therapy, Idursulfase, Rare disease, Registry, Filipino

## Abstract

**Background:**

Mucopolysaccharidosis type II (MPS II; Hunter syndrome) is an X-linked multisystem disorder characterized by glycosaminoglycan (GAG) accumulation, caused by a deficiency of iduronate-2-sulfatase (I2S). Enzyme replacement therapy (ERT) with recombinant idursulfase (IDS), the standard of care, was started in the Philippines in 2017. This study reviewed the clinical outcomes in idursulfase-treated and untreated Filipino MPS II patients who were included in the local Lysosomal Storage Disease (LSD) registry of the Institute of Human Genetics-National Institutes of Health (IHG-NIH) from January 1999 to December 2019.

**Methods:**

A retrospective audit of records of MPS II patients listed in the registry was done. Qualified patients were divided into two cohorts: idursulfase-treated group (patients on enzyme replacement therapy, ERT, for ≥ 6 months) and untreated group. Baseline characteristics, including demographic data, biochemical results, neurocognitive classification, respiratory involvement, mortality, and adverse events, were recorded. Height, weight, cardiac pathology, liver and spleen sizes, six-minute walking test (6MWT), joint mobility, were determined at baseline and at year 1 and 2 of follow up.

**Results:**

Forty male patients were included in this review, with only 8 receiving ERT since 2017. The mean age at diagnosis was 6.99 years (SD 4.15; 0.75–20) and mean age at start of ERT was 14.03 years (SD 7.1; 4–21.5), more delayed than previous reports. Eighty percent have early progressive phenotype which was higher than reported average. The early growth pattern differed in our Filipino cohort, but was followed by the expected slowed growth in later years. Improvements in the following endpoints were observed in the treated cohort: height and weight, cardiac disease, liver and spleen sizes, and joint mobility. There were also positive effects on respiratory involvement and mortality rate. Adverse events were consistent with previous reports.

**Conclusions:**

ERT is generally well tolerated and effective in reducing GAG storage and improving clinical endpoints among our Filipino MPS II patients. In untreated patients, typical disease progression was observed.

## Introduction

Mucopolysaccharidosis type II (MPS II; also known as Hunter syndrome) is an X-linked multisystem disorder characterized by glycosaminoglycan (GAG) accumulation, caused by a deficiency of iduronate-2-sulfatase (I2S) [1]. The enzyme deficiency in MPS II leads to primary accumulation of dermatan sulfate (DS) and heparan sulfate (HS) [2]. The buildup of these GAGs accounts for heterogenous somatic and neurologic manifestations of the disease [3].

The clinical manifestations of MPS II, including age of onset, disease severity, and rate of progression vary significantly among affected males. Until recently, phenotypes are designated as "slowly progressive" or "early progressive", depending on the presence or absence of CNS involvement, respectively [1]. In those with early progressive disease, there is progressive cognitive deterioration, progressive airway and cardiac disease that usually result in death in the first or second decade of life. While in those with slowly progressive disease, the CNS is minimally or is not affected, although the effect of GAG accumulation on other organ systems may be the same as in early progressive types. Other clinical features present in both phenotypes include: short stature, coarse facies, macrocephaly with or without communicating hydrocephalus, macroglossia, hoarse voice, conductive and sensorineural hearing loss, frequent sinus and ear infections, umbilical hernia, hepatosplenomegaly, dysostosis multiplex, spinal stenosis, joint contractures and carpal tunnel syndrome. [1]

A multidisciplinary approach to treatment and symptomatic care remain to be the most important aspects of MPS management. However, disease-modifying strategies are available and include enzyme replacement therapy (ERT) and hematopoietic stem cell transplantation (HSCT) [2]. Of the two, ERT with recombinant idursulfase (IDS) is currently the recommended treatment strategy that is used routinely in clinical practice. In order to assess the efficacy of treatment with IDS and to monitor the response to ERT, it has been suggested to continuously evaluate different clinical, laboratory, and instrumental endpoints that may include: urinary GAGs excretion, six-minute walking test (6MWT), forced vital capacity (FVC), liver and spleen volumes, and joint mobility [4]. A series of studies have consistently demonstrated somatic improvements with ERT that consequently result in a better quality of life for some patients, [5–8] as well as improved survival [9]. Idursulfase has also been shown to be generally well tolerated, with a safety profile similar to that reported for ERT in patients with other mucopolysaccharidoses [1]. The main limitation of therapy, however, is the inability of IDS to cross the blood–brain barrier rendering it ineffective against neurological symptoms [4].

The global incidence of MPS II is 1:100,000–170,000 male births. Hunter syndrome is the most prevalent of all MPS patients in the Philippines, comprising around 84%, [10] similar in other East Asian regions [3, 11]. As of writing, 59 MPS II patients have been recorded in the Lysosomal Storage Disease (LSD) registry of the Institute of Human Genetics-National Institutes of Health (IHG-NIH), the only institution in the Philippines providing genetic services. The listing was started in 1999 to provide an updated census of Filipinos diagnosed with any of the LSDs that later evolved into a registry that included baseline information and outcomes of patients with these conditions. However, this registry is limited to patients reported to the Philippine General Hospital (PGH) and/or IHG-NIH. This was patterned after the Hunter Outcome Survey (HOS), which was established in 2005, by a global scientific advisory board comprised of physicians experienced in MPS II. For rare diseases, a large, multicenter, observational registry is useful in collecting long-term data on the natural history of MPS II and the efficacy and safety of treatment from a broad population of patients [1].

It has now been a more than a decade since ERT with intravenous IDS has been approved and used as standard of care. Locally, ERT for a limited number of Filipino patients was started in 2017. Though it is regarded as the standard of care, IDS is very expensive. It costs approximately Php 1 to 1.5 million per month to treat a 15 to 30 kg patient. This is the main reason why continuous ERT could be started on only eight of the 40 patients in this review. It also requires weekly injections causing a disruption in the everyday lives of patients and their caregivers. The aim of the study is to review the clinical outcomes of idursulfase-treated and untreated Filipino patients with MPS II who were included in the LSD Registry from January 1999 to December 2019. By looking at these outcomes, we could have a better understanding of its effects and safety among our Filipino cohort. Long-term follow up of these patients will also give a more comprehensive look into its disease progression.

## Methods

This is a retrospective descriptive study of MPS II patients diagnosed at the Philippine General Hospital (PGH), University of the Philippines Manila (UPM) and/or at the IHG-NIH who were subsequently listed in the LSD Registry from January 1999 to December 2019. Waiver of informed consent was applied since the retrospective method of data collection would entail not more than minimal risk. The study protocol was approved by UPM Research Ethics Board (2020-108-01).

An audit of the records from the Registry was supplemented by patient charts and included only biochemically-confirmed MPS II cases with at least one follow-up at the PGH outpatient department, in-patient wards, or MPS multidisciplinary clinic. They were divided into two cohorts as follows: (1) idursulfase-treated group included patients who have been receiving ERT with recombinant IDS at 0.5 mg/kg administered weekly and continuously for at least 6 months and (2) Untreated group who have not received IDS at any point or have received ERT for less than 6 months.

Age at onset of symptoms, age at diagnosis, urinary GAG concentration at diagnosis, plasma enzyme assay level, age at start of treatment, length of time on treatment, cognitive impairment (diagnosed and classified at any time by a Developmental Pediatrician based on Diagnostic and Statistical Manual for Mental Disorders 5, or DSM-5), respiratory disease, deceased, age at death, and cause of death were collected from the registry by the primary investigator. Further, neurodevelopmental disorder was classified as either Global Developmental Delay (GDD) or Intellectual Disability (ID) according to DSM-5 criteria (GDD: significant delay in at least two developmental domains in children < 5 years old; ID: deficits in intellectual and adaptive functioning reserved for individuals over the age of 5 years).

### Clinical parameters

Clinical parameters in the IDS-treated group were recorded at baseline and at year 1 and 2 of treatment. A post-baseline measurement was defined as the value recorded closest to the first and second year from treatment initiation, within 6 months either side of the date. For the untreated cohort, post-baseline measurement was defined as the value recorded closest to the first and second year from diagnosis, within 6 months either side of the date.

#### Growth

Since growth is age-dependent, data on height (in cm) and weight (in kg), with corresponding z scores, and absolute change from baseline were stratified and analyzed within three age groups (Group 1: aged < 6 years, Group 2: 6–10 years, and Group 3: > 10 years). Ages 6 and 10 years old were chosen as age cutoffs at the start of ERT (for treated group) or age at diagnosis (for the untreated group) because growth tend to be faster during the early years, followed by slowed growth starting at 8 years and beyond [12].

#### Cardiac pathology

Cardiac pathology, as evident on echocardiographic data, were recorded. The presence of left ventricular hypertrophy (LVH) was based on echocardiogram findings and defined by left ventricular mass index (LVMI) calculated as the left ventricular mass normalized for body surface area according to the recommendations of the American Society of Echocardiography (ASE). The diagnosis of LVH is attained when the LVMI is ≥ 102 g/m^2^ [6].

#### Liver and spleen size

Since sonographic measurements of liver and spleen were not routinely done, sizes were measured in cm by palpation according to standard clinical practice.

#### Six-minute walking test (6MWT)

This was measured in meters as the distance that the patient was able cover in six minutes.

#### Joint mobility

Joint range of motion (ROM) of shoulder, elbow, wrist, hip, knee and ankle mobility in degrees were recorded using a goniometer appropriately aligned using the proper landmarks of a specific joint. ROM of the extremities were done in a cephalocaudal manner of patients that were positioned while in supine or seated, depending on the position more tolerated by the patient. The ROM was performed by a rehabilitation medicine resident physician or by a licensed physical therapist present during different clinic schedules.

#### Safety

Safety profile was determined as the proportion of patients reported to have any adverse events (AEs) and recorded as either infusion-related reactions (IRR), defined as AEs assessed to be drug-related and occurring within 24 h of the infusion, or not related (Non-IRR).

### Biochemical studies

LSD assay for all 40 patients were sent to the laboratory of the Department of Medical Genetics, National Taiwan University Hospital. Confirmed cases are patients with either an enzyme activity measurement and/or quantitative urine GAGs. While we recognize the importance of doing both tests, due to financial constraints we are only able to do one or the other in some patients (one patient without plasma I2S level, three patients without urine GAGs measurements). For these patients, we had to rely on clinical judgement, and given the high index of suspicion supported by one of the laboratories, we made the diagnosis. Plasma I2S activity was measured in using the 4-methylumbelliferone (4-MU) fluorometric enzyme assay. The concentration of urinary glycosaminoglycans was measured using the Dimethylene Blue (DMB) assay with results compared with the established reference ranges per age group of urinary GAGs per grams of creatinine.

### Data analysis

Descriptive statistics were used to summarize demographic characteristics of the patients.

Categorical variables (presenting manifestations, respiratory symptoms, neurocognitive classification, cardiac disease, mortality, and adverse events) were summarized as proportions and frequency distributions were obtained. Continuous variables (height, weight, LVMI, extent of hepatosplenomegaly, 6MWT, joint ROM, and biochemical results) were summarized as means, standard deviation and range (minimum and maximum values). This was done separately for treated and untreated patients, at baseline, year 1, and year 2 of follow-up.

For height and weight, data was analyzed according to three groups based on the age at start of ERT or at time of diagnosis for the untreated group (Group 1: aged < 6 years, Group 2: 6–10 years, and Group 3: > 10 years). Analysis was done at baseline, year 1 and 2 of follow up.

## Results

As of December 2019, there were 59 Hunter syndrome patients listed in the Philippine LSD Registry, 40 of whom fulfilled the inclusion criteria. Eight out of the 40 are on regular and continuous ERT with recombinant IDS (Elaprase®, Shire Human Genetic Therapies, Inc., Cambridge, MA, USA). Six of the patients on continuous therapy satisfied the criteria for ERT based on the Philippine management guidelines for MPS II—documented biochemical diagnosis, without severe cognitive impairment, and with at least one clinical manifestation that is still deemed treatable and not too far advanced to be addressed by ERT. While two more patients with severe cognitive impairment were started on IDS based on the inclusion criteria delineated by a multi-center experimental study there were enrolled in. At the time of review, 27 of 40 patients are alive.

### Demographic characteristics

A total of 40 male patients from 29 non-consanguineous families were included in this review. They were all of Filipino descent except for one patient under the treatment group who was adopted out of the family, born to a Filipina mother and a Caucasian father. The mean age at onset of symptoms was 2.28 years (SD 1.70; range 0.5–6). The average age at the time of diagnosis was 6.99 years (SD 4.15) with time to diagnosis of 0.75 of a year to as late as 20 years after onset of symptoms. Among the 27 patients who were still alive, their mean age was 13.6 years at the time of review (SD 6.4; range 4.42 to 30.33).

Among the 40 patients, 9 were initially started on ERT. However one patient withdrew after the 6th session following an anaphylactic reaction. Eight patients were classified under the treatment group as they continue to receive weekly ERT for at least 6 months. The mean age at the start of ERT was 14.03 years old (SD 7.1; 4–21.5), with mean delay of 9 years (SD 6.68; 0.25–20.66) from time of diagnosis to start of ERT. Since ERT was started in our country in 2017, the patients have been receiving weekly therapy for mean of 21.12 months (SD 8.7; 10–32) in duration.

During the initial evaluation, the most common presenting manifestations elicited among the patients were joint contractures in 12 (30.77%) and recurrent respiratory tract infections in 11 (28.2%) (Table 1).

**Table 1:** Presenting clinical manifestations among Filipino Hunter syndrome patients on initial evaluation

Presenting manifestation	Number (n = 40)	Percentage (%)
Joint contractures	12	30
Recurrent respiratory tract infections	11	27.5
Coarse facies	9	22.5
Developmental delay	9	22.5
Inguinal hernia	4	10
Hepatosplenomegaly and/or abdominal enlargement	3	7.5
Short stature	1	2.5

Note: Ten patients presented with two different symptoms and were counted separately

### Clinical outcomes

#### Height

Eleven patients were classified under group 1 (treated = 2 patients, mean age at starting ERT = 4.7 years; untreated = 9, mean age at diagnosis = 3.39 years). The mean height at baseline was 100.55 cm, ranging from 80 to 111 cm (SD 9.74). Only 1 patient was classified as stunted (WHO z-score below -2, Table 2) at the onset, coming from the treated cohort. At the end of the 2-year follow up period, the mean height was 110.83 cm (SD 7.33; 104.4–130), with an overall increase in height by 10.28 cm. Two patients from the untreated group were reclassified as stunted, bringing the total to 3 (27.3%) stunted patients at end of year 2 (Table 2).

**Table 2:** Distribution of height z-scores of treated and untreated MPS II patients at baseline, year 1, and year 2 of follow up as classified in to age groups

	All (n = 11)	Treated (n = 2)	Untreated (n = 9)
**Group 1**			
Baseline			
Stunted (z < − 2) %	1 9%	1 50%	0
Normal (z < − 1 to > + 2) %	10 91%	1 50%	9 100%
Tall (z > + 3) %	0	0	0
Year 1			
Stunted (z < − 2) %	3 27.3%	1 50%	2 22.22%
Normal (z < − 1 to > + 2) %	8 72.7%	1 50%	7 77.8%
Tall (z > + 3) %	0	0	0
Year 2			
Stunted (z < − 2) %	3 27.3%	1 50%	2 22.22%
Normal (z < − 1 to > + 2) %	8 72.7%	1 50%	7 77.8%
Tall (z > + 3) %	0	0	0
**Group 2**			
Baseline (n = 16; Treated = 1; Untreated = 15)			
Stunted (z < − 2) %	11 68.75%	1	10 66.67%
Normal (z < − 1 to > + 2) %	5 31.25%	0	5 33.33%
Tall (z > + 3) %	0	0	0
Year 1 (n = 13; Treated = 1; Untreated = 12)			
Stunted (z < − 2) %	10 77%	1	9 75%
Normal (z < − 1 to > + 2) %	3 23%	0	3 25%
Tall (z > + 3) %	0	0	0
Year 2 (n = 10; Treated = 0; Untreated = 10)			
Stunted (z < − 2) %	7 70%	NA	7 70%
Normal (z < − 1 to > + 2) %	3 30%	NA	3 30%
Tall (z > + 3) %	0	NA	0
**Group 3**			
Baseline (n = 13; Treated = 5; Untreated = 8)			
Stunted (z < − 2) %	13 100%	5 100%	8 100%
Normal (z < − 1 to > + 2) %	0	0	0
Tall (z > + 3) %	0	0	0
Year 1 (n = 11; Treated = 5; Untreated = 6)			
Stunted (z < − 2) %	11 100%	5 100%	6 100%
Normal (z < − 1 to > + 2) %	0	0	0
Tall (z > + 3) %	0	0	0
Year 2 (n = 7; Treated = 4; Untreated = 3)			
Stunted (z < − 2) %	7 100%	4 100%	3 100%
Normal (z < − 1 to > + 2) %	0	0	0
Tall (z > + 3) %	0	0	0

Group 1: aged < 6 years at start of ERT or at time of diagnosis; Group 2: aged 6–10 years at start of ERT or at time of diagnosis; Group 3: aged > 10 years at start of ERT or at time of diagnosis

Sixteen patients were classified under group 2. One patient was treated. ERT was started when the child was 10 years old. The child was stunted. For the 15 untreated patients, the mean age at diagnosis is 7.67 years, and the mean height at baseline was 109.34 cm (SD 9.37; 96–127). Eleven of the 16 (68.75%) patients were considered stunted at the outset. At the end of year 2, only 10 patients have data. The mean height was 115.43 cm (SD 12.3; 99–134) with an overall increase in height by 3.23 cm. The percentage of stunted patients (70%) was comparable with baseline data (Table 2).

Thirteen patients were classified under group 3 (treated = 5, mean age at starting ERT = 17 years; untreated = 8, mean age at diagnosis = 15.25 years). The mean height at baseline was 116.68 cm (SD 13.91; 95–143.8). All 13 patients in this group were classified as stunted at the outset (Table 2). After the 2-year follow up period, the mean height for all was 125.57 cm (SD 13.87; 110–144) with an overall increase in height by 5.59 cm.

**Fig. 1:**
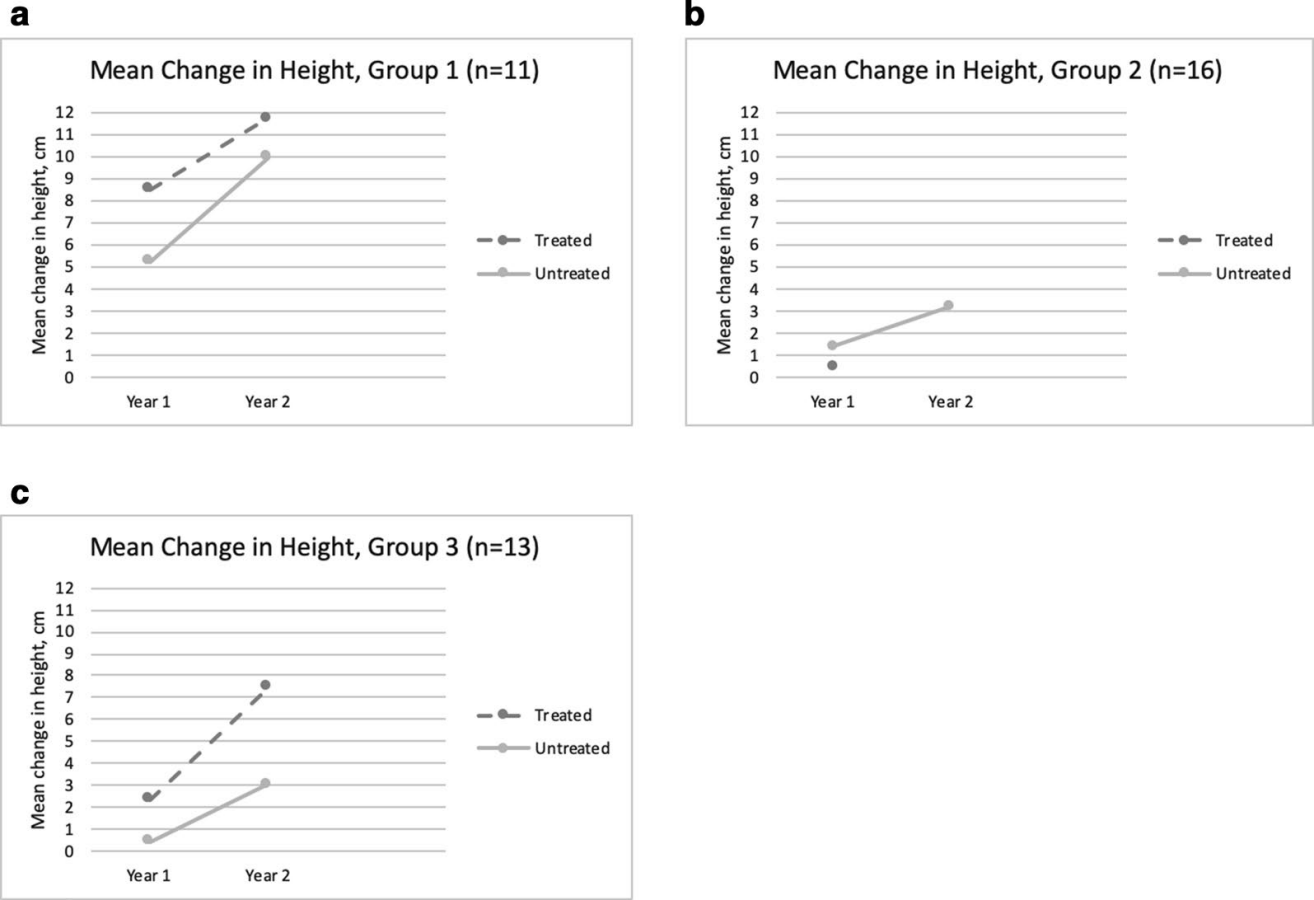
Mean annual change in height for treated and untreated MPS II patients at year 1 and year 2 of follow up as classified in to age groups. **a** Group 1: aged < 6 years at start of ERT or at time of diagnosis, **b** Group 2: aged 6–10 years at start of ERT or at time of diagnosis. **c** Group 3: aged > 10 years at start of ERT or at time of diagnosis

**Fig. 2:**
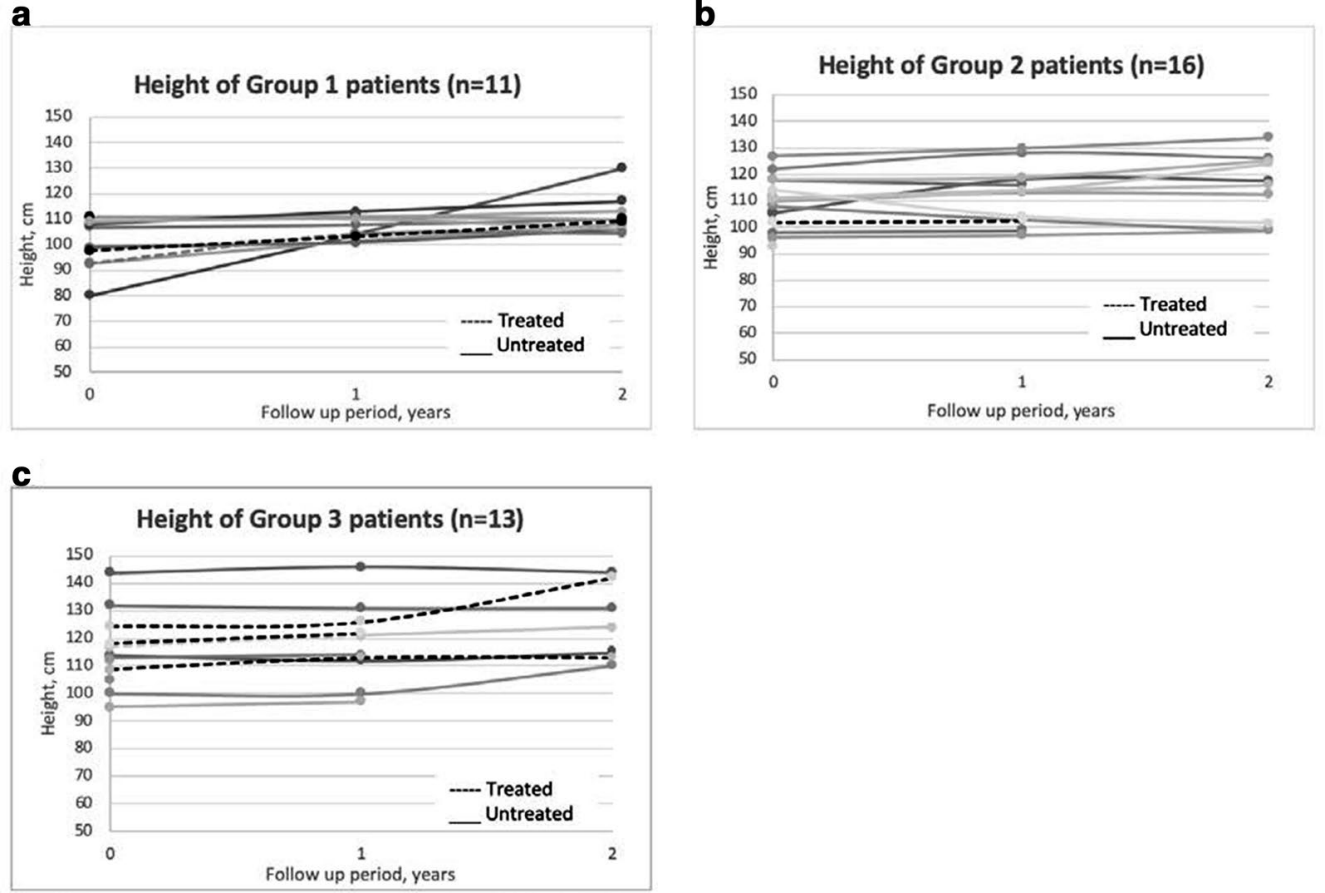
Height of treated and untreated MPS II patients at baseline (0) year 1 and year 2 of follow up as classified in to age groups. **a** Group 1: aged < 6 years at start of ERT or at time of diagnosis, **b** Group 2: aged 6–10 years at start of ERT or at time of diagnosis. **c** Group 3: aged > 10 years at start of ERT or at time of diagnosis

At the end of the 2-year follow up period, as observed in groups 1 and 3 (Fig. 1), there was a greater increase in height in the treated group compared to the untreated group, with higher height increase noted in the younger group (group 1) than in the older cohort (group 3). Further, none of the treated patients were shorter than their baseline height across all age-stratified groups (Fig. 2), as compared with the untreated group wherein 5 patients were recorded to be shorter by an average of 5.3 cm than their baseline height. As the patients age, there was a notable increase in the percentage of stunted patients, to such an extent that all patients in Group 3 were stunted (Table 2).

#### Weight

The distribution of patients across the age-stratified groups were similar with the height data. In group 1 patients, mean weight at baseline was 18.86 kg, ranging from 11.3 to 23.4 kg (SD 3.36). None of the patients were classified as underweight at the onset, (WHO z-score below -2, Table 3). At the end of 2-year follow up period, the mean weight was 23.08 kg (SD 2.76; 20.5–30.3) with an overall increase in weight by 4.22 kg. Only 1 out of 11 (9%) patients was reclassified as underweight at end of year 2, from the untreated cohort (Table 3).

**Table 3:** Distribution of weight z-scores of treated and untreated MPS II patients at baseline, year 1, and year 2 of follow up as classified in to age groups

	All (n = 11)	Treated (n = 2)	Untreated (n = 9)
**Group 1**			
Baseline			
Underweight (z < − 2) %	0	0	0
Normal (z < − 1 to + 2) %	9 81.9%	2 100%	7 77.78%
Overweight (z > + 2) %	2 18.1%	0	2 22.22%
Year 1			
Underweight (z < − 2) %	1 9%	0	1 11.11%
Normal (z < − 1 to + 2) %	8 72.72	2 100%	6 66.67%
Overweight (z > + 2) %	2 18.18	0	2 22.22
Year 2			
Underweight (z < − 2) %	1 9%	0	1 11.11%
Normal (z < − 1 to + 2) %	9 81.9%	2 100%	7 77.78%
Overweight (z > + 2) %	1 9%	0	1 11.11%
**Group 2**			
Baseline (n = 16; Treated = 1; Untreated = 15)			
Underweight (z < − 2) %	3 18.75%	1	2 13.33%
Normal (z < − 1 to + 2) %	13 81.25%	0	13 86.67%
Overweight (z > + 2) %	0	0	0
Year 1 (n = 13; Treated = 1; Untreated = 12)			
Underweight (z < − 2) %	4 30.77%	1	3 25%
Normal (z < − 1 to + 2) %	9 69.23%	0	9 75%
Overweight (z > + 2) %	0	0	0
Year 2 (n = 11; Treated = 0; Untreated = 11)			
Underweight (z < − 2) %	5 45.45%	NA	5 45.45%
Normal (z < − 1 to + 2) %	6 54.54%	NA	6 54.54%
Overweight (z > + 2) %	0	NA	0
**Group 3**			
Baseline (n = 13; Treated = 5; Untreated = 8)			
Underweight (z < − 2) %	10 76.9%	4 80%	6 75%
Normal (z < − 1 to + 2) %	3 23.2%	1 20%	2 25%
Overweight (z > + 2) %	0	0	0
Year 1 (n = 12; Treated = 5; Untreated = 7)			
Underweight (z < − 2) %	11 91.67%	4 80%	7 100%
Normal (z < − 1 to + 2) %	1 12.5%	1 20%	0
Overweight (z > + 2) %	0	0	0
Year 2 (n = 8; Treated = 4; Untreated = 4)			
Underweight (z < − 2) %	7 87.5%	3 75%	4 100%
Normal (z < − 1 to + 2) %	1 12.5%	1 25%	0
Overweight (z > + 2) %	0	0	0

Group 1: aged < 6 years at start of ERT or at time of diagnosis; Group 2: aged 6–10 years at start of ERT or at time of diagnosis; Group 3: aged > 10 years at start of ERT or at time of diagnosis

Sixteen patients were classified under group 2, and the mean weight at baseline was 22.26 kg (SD 3.86; 18–29.4). Three of the 16 (18.75%) patients were considered underweight at the outset, including the only patient from the treatment group. At the end of year 2, only 11 untreated patients have data. The mean weight was 25.05 kg (SD 4,74; 16.7–33.18) with an overall increase in weight by 2 kg. However there was a greater percentage (45.5%) of underweight patients (Table 3).

Thirteen patients were classified under group 3, and the mean weight at baseline was 28.26 kg (SD 7.13; 17.2–44.5). Eleven of the 13 (84.62%) were classified as underweight at the outset. After the 2-year follow up period, the mean weight was 33.03 (10.7; 17–50.8) with an overall increase in weight by 3.11 kg. There was slightly greater percentage (87.5%) of underweight patients overall, with all untreated patients being underweight by the end of year 2 (Table 3).

**Fig. 3:**
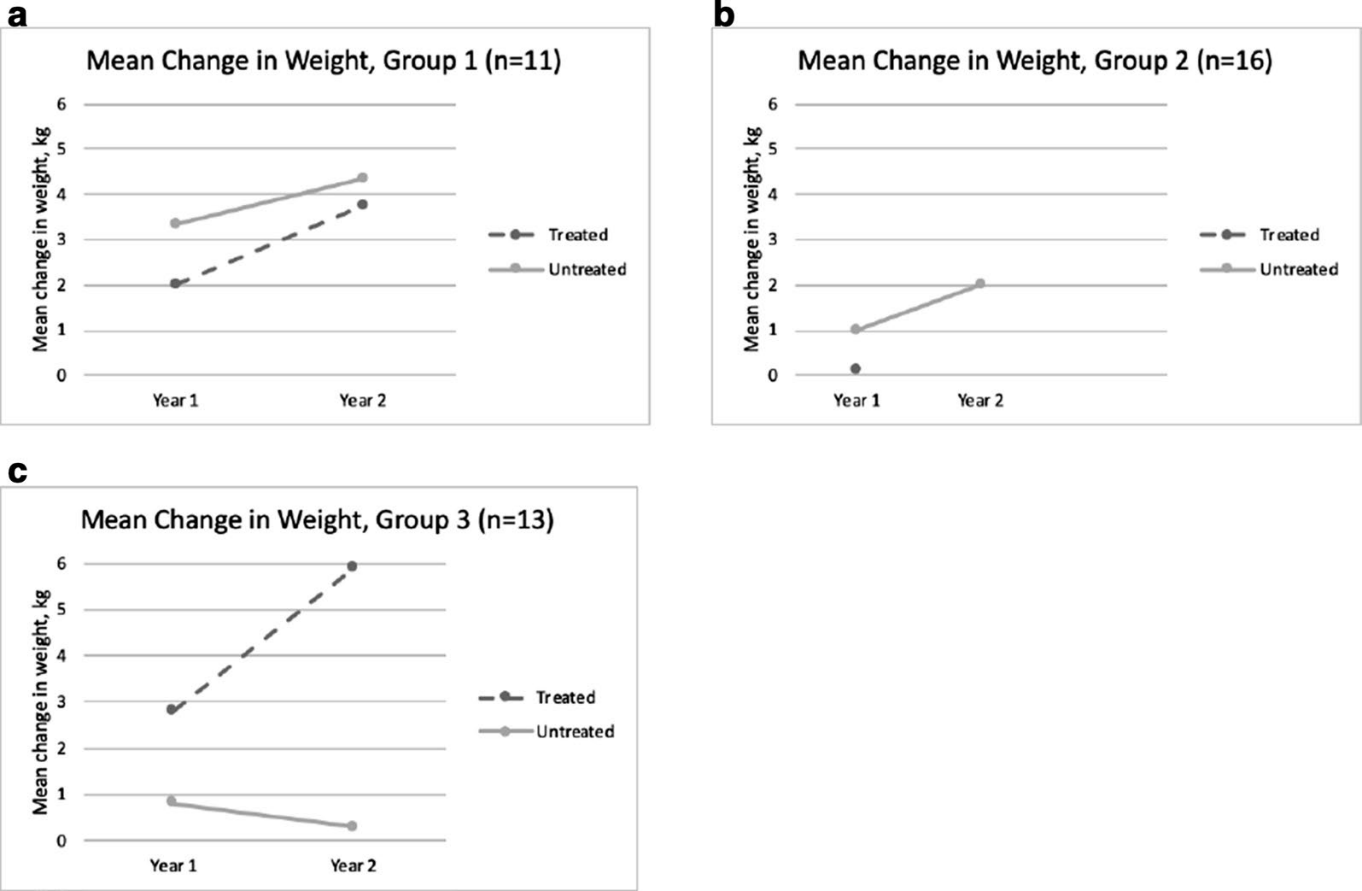
Mean annual change in weight for treated and untreated MPS II patients at year 1 and year 2 of follow up as classified in to age groups. **a** Group 1: aged < 6 years at start of ERT or at time of diagnosis, **b** Group 2: aged 6–10 years at start of ERT or at time of diagnosis. **c** Group 3: aged > 10 years at start of ERT or at time of diagnosis

**Fig. 4:**
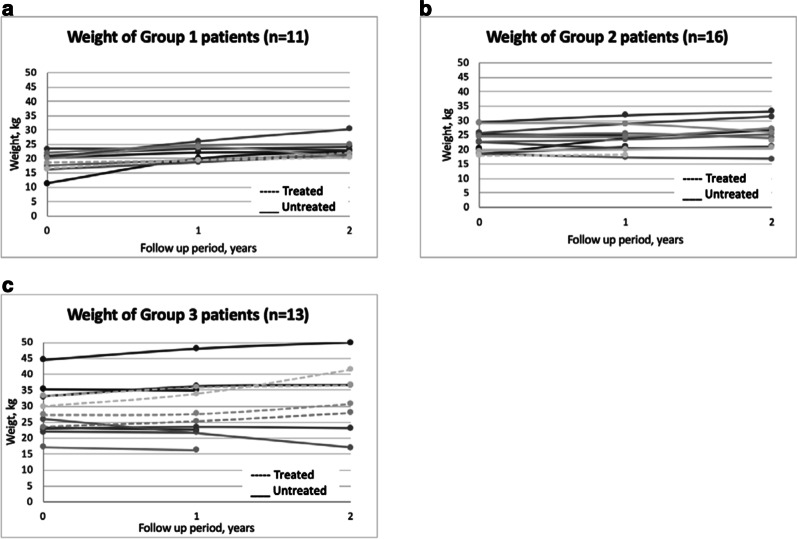
Weight of treated and untreated MPS II patients at baseline (0) year 1 and year 2 of follow up as classified in to age groups. **a** Group 1: aged < 6 years at start of ERT or at time of diagnosis, **b** Group 2: aged 6–10 years at start of ERT or at time of diagnosis. **c** Group 3: aged > 10 years at start of ERT or at time of diagnosis

At the end of the 2-year follow up period, there was a greater interval increase in weight in the untreated cohort under group 1, but the reverse was seen in group 3 patients (Fig. 3). All treated patients experienced continued weight gain over the 2-year observation period, while 11 untreated patients had weight loss recorded at least once during their follow up (Fig. 4). Lastly, there was an increasing percentage of underweight patients annually in the untreated group, to such an extent that all patients in the group 3 were underweight at the end of year 1 and 2 (Table 3).

#### Neurocognitive classification

Based on the recent classification according to phenotype, [1] 80% of the patients (32/40) presented with early progressive course, or severe, during the initial assessment. Twenty percent (8/40) fall under the slowly progressive, or attenuated, form of the disease, with only 1 patient with normal neurocognitive status, coming from the treated cohort. Among the 8 treated patients, 4 patients (50%) have an early progressive form and the other half were assessed to have a slowly progressive course. Baseline neurocognitive classification according to DSM-5 and their distribution are shown in Table 4.

**Table 4:** Baseline phenotypic classification according to severity and distribution according to developmental assessment and treatment group

	Classification (n = 40) (%)	Developmental assessment	Treated (n = 8) (%)	Untreated (n = 32) (%)	Total (n = 40) (%)
Early progressive	32 (80%)	GDD	2 (25%)	6 (18.75%)	8 (20%)
		ID, moderate	0	2 (6.25%)	2 (5%)
		ID, severe	0	8 (25%)	8 (20%)
		ID, unclassified	2 (25%)	12 (37.5%)	14 (35%)
Slowly progressive	8 (20%)	ID, mild	3 (37.5%)	4 (12.5%)	7 (17.5%)
		No cognitive impairment	1 (12.5%)	0	1 (2.5)

#### Respiratory

During the follow-up period, a total of 31/40 patients (77.5%) developed respiratory involvement (Table 5). A greater percentage of untreated patients had respiratory symptoms (81.25%) as compared with the treated cohort (62.5%). The percentage of CPAP-dependent patients are comparable in both cohorts. None of the treated patients necessitated tracheostomy thus far.

**Table 5:** Respiratory involvement and diagnosis among the treated and untreated cohorts recorded throughout the follow up period

	Respiratory involvement	Bronchial asthma	OSA*	CPAP dependent	Tracheostomy
Treated (n = 8)	5 (62.5%)	4 (50%)	4 (50%)	1 (12.5%)	0
Untreated (n = 32)	26 (81.25%)	6 (18.75%)	20 (62.5%)	4 (12.5%)	2 (6.25%)
Total (n = 40)	31 (77.5%)	10 (25%)	24 (60%)	5 (12.5%)	2 (5%)

*OSA: obstructive sleep apnea

#### Cardiac disease

Baseline cardiac data was available from 23 patients (treated = 4, untreated = 19). Majority (15/23; 65.2%) had evidence of cardiac disease on 2D echocardiogram, including valvular disease in 13 (56.52%) and/or chamber enlargement in 9 (39.13%). The most commonly involved valves were the mitral and atrial valves; while the left ventricle and atrium were the most commonly enlarged chambers. At the end of the 2-year follow up period, data from 11 patients was available (treated = 3, untreated = 8), showing a greater percentage (81.8%) of patients with cardiac involvement. However, following 2 years of therapy, there was an overall improvement in cardiac profiles of the treated cohort. Two of the 3 (66.67%) patients had valvular involvement, compared to 100% involvement at baseline. In addition, in two treated siblings, there was improvement in their cardiac dimensions as both achieved normal chambers, consistent with decrease in their LVMI (mean decrease of 25 g/m^2^). None of the treated patients were found to have cardiac hypertrophy after 2 years. While in the untreated cohort, the natural progression of cardiac disease was observed in the majority. Compared with baseline of 47.37% (9/19), 75% of patients (6/8) had valvular involvement. At baseline, 36.84% of untreated patients (7/19) had chamber enlargement, which increased to 75% (6/8) after 2 years. Among the untreated patients with complete data throughout the follow up period, 1 remained to have normal cardiac findings, 1 had further depression in systolic function with concomitant decrease in ejection fraction, 2 developed chamber enlargement, 1 had progression in both valvular disease and cardiac hypertrophy, and 3 had the same findings as baseline.

**Fig. 5:**
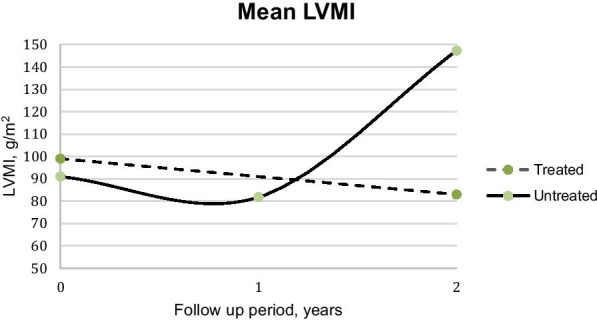
Mean LVMI in g/m^2^ as measured by 2d echocardiogram, according to treatment group. (LVH: LVMI ≥ 102 g/m^2^) [6]. Baseline n = 17 (Treated = 5, Untreated = 12), year 1 n = 5 (treated = no information available, untreated = 5), and year 2 n = 6 (treated = 3, untreated = 3)

As in the study by Muenzer, LVH is defined here as LVMI of ≥ 102 g/m^2^ [6]. In this study cohort, the mean baseline LVMI for all was 92 g/m^2^ (SD 26; 61–162). Overall, there was progression in the mean LVMI after 2 years to 115.17 g/m^2^ (SD 26; 61–162). However, this largely came from the untreated cohort wherein LVMI progressed to 147.33 g/m^2^ (SD 56.15; 111–212) after 2 years, in contrast to the mean LVMI for the treated cohort which improved to 83 g/m^2^ (SD 12; 71–95, Fig. 5).

#### Hepatosplenomegaly

Among the 36 patients with baseline data, 21 (58.33%) had liver enlargement with mean measurement of 4.79 cm below RCM (SD 2.4; 2–12). In the treatment group, 7/8 patients (87.5%) had hepatomegaly (mean 4.43 cm below RCM), while 50% of the untreated cohort (14/28) had liver edge palpable up to mean of 4.96 cm from RCM. At the end of 2-year follow up, there was resolution of hepatomegaly in all treated patients (3 of which resolved after 1 year of therapy). In the untreated group, 15/16 patients (93.75%) remained to have hepatomegaly with mean of 4.78 cm below RCM. There was also progression in liver size by 4.71 cm from baseline noted in 9 untreated patients.

For spleen size, baseline data was available for 34 patients, with only 8 patients (23.53%) presenting with splenomegaly with mean measurement of 4.44 cm below LCM. Two of the 8 (25%) treated patients and 6/26 (23%) of untreated patients had baseline splenomegaly. After 1 year of ERT, there was resolution of splenomegaly in 1 treated patient, while the remaining patient’s spleen was palpable up to 1 cm below LCM only. This resolved by the end of second year of ERT, so that none of the treated patients had splenomegaly at the end of the follow up period. In the untreated group, a greater percentage (50%) of patients had palpable spleen up to 6.67 cm, with progression in size by 4.83 cm in 6 patients.

#### 6MWT

Of the 40 patients, 13 were non-ambulatory, wheelchair-bound, all coming from untreated cohort. From the 27 ambulatory patients, only 2 treated patients had complete data throughout the 2-year follow-up period. One patient was seen to have a decline in the tolerated distance by 91.44 m from baseline to year 2 of follow up. While the other patient showed an improvement in the distance covered by 172 m after 2 years of treatment. Nine untreated patients had data collected only on their 2nd year follow up, which showed below average distance covered.

#### Joint mobility

Thirteen out of the 40 patients had baseline data only and values indicate already existing joint limitations (Table 6). Only 7 patients had complete ROM data throughout the 2-year follow up period. There was an average of 10–20 degrees improvement of ROM mainly on the shoulders, hips and knees; with an average of 10–20 degrees worsening of joint mobility for elbow flexion and extension, ankle dorsiflexion and plantar flexion. In all, lower extremity joints were more likely to worsen compared to the upper extremity joints.

**Table 6:** Mean change in ROM from baseline to 2 years for treated and untreated groups

	Treated (n = 8)			Untreated (n = 32)		
	Baseline (mean °)	Year 2 (mean °)	Change* (°)	Baseline (mean °)	Year 2 (mean °)	Change* (°)
Shoulder Flexion	111	101	− 10	90.38	86.5	− 3.88
Shoulder Extension	53.75	61.25	7.5	100	90	− 10
Shoulder Abduction	99.13	98.4	− 0.725	85.63	87.78	2.15
Shoulder Internal Rotation	55	67	12	40	30	− 10
Shoulder External Rotation	52.5	59.25	6.75	35	40	5
Elbow Flexion	93.13	89.5	− 3.63	109.17	104.83	− 4.33
Elbow Extension	111	− 45	66	− 122.5	− 112.5	10
Elbow Supination	10	10	0	80	80	0
Elbow Pronation	35	55	20	80	62.5	− 17.5
Wrist Flexion	46.2	32.5	− 13.7	33.75	6.25	− 27.5
Wrist Extension	22.9	27.5	4.6	25	9.13	− 15.88
Wrist Ulnar Deviation	No information	No information	No information	25	2.5	− 22.5
Wrist Radial Deviation	25	20	− 5	No information	No information	No information
Hip Flexion	126.08	108.83	− 17.25	No information	No information	No information
Hip Extension	8.75	12.25	3.5	No information	No information	No information
Hip Abduction	36.25	46.75	10.5	No information	No information	No information
Hip Internal Rotation	42.5	39.5	− 3	No information	No information	No information
Hip External Rotation	50	50.25	0.25	No information	No information	No information
Knee Flexion	119.13	122.75	3.63	96.28	127.08	30.81
Knee Extension	109.5	− 122.5	− 13	No information	No information	No information
Ankle Dorsiflexion	1.67	4.17	2.5	5	8.13	3.13
Ankle Plantarflexion	37.33	38.38	1.04	40	27.5	− 12.5
Ankle Eversion	5.5	3.75	− 1.75	No information	No information	No information
Ankle Inversion	7.5	14	6.5	No information	No information	No information

^*^Negative change in ROM: more limited joint mobility; positive change in ROM: improvement in mobility overtime

For the treated group, there was improvement in shoulder extension and internal rotation, elbow extension and pronation, wrist flexion and extension, and ankle plantarflexion, but with more limited knee flexion after 2 years of ERT. In the untreated cohort, improvement in joint mobility was seen in shoulder abduction, shoulder external rotation, knee flexion, and ankle dorsiflexion after 2 years. There was a decrease in mean ROM in shoulder flexion and extension, shoulder internal rotation, elbow flexion, elbow pronation, wrist flexion and extension, and ankle plantar flexion, indicating worsening of contractures in the untreated patients.

#### Biochemical profile

In 34 samples out of the 40 patients, I2S was measured as nmol/mg plasma/4h and the mean enzyme level was 0.53 nmol/mg plasma/4 h (SD 0.56; 0.01–2.34; NV: > 167 nmol/mg plasma/4 h), while 5 samples were measured as uM/hr I2S with mean of 0.12 (SD 0.03; 0.09–0.17; NV > 4.45 uM/hr). The mean urinary GAG concentration for the 27 of the 40 patients was 525.96 mg/g creatinine (SD 204.35; 126.1–911.46; NV: Age ≤ 1 year: 20.26–312.38; 1 to ≤ 3 years: 19.97–110.53; 3 to ≤ 5 years: 10.74 -112.02; ≥ 5 years: 10.77–77.5 mg/g creatinine). The urine GAGs were reported in separate components of HS and DS in 10 patients, with the following results: mean urinary HS of 35.87 ng/ug crea (SD 17.15; 14.85–58.4; NV: Age 0–2 years: 0.65–3.51; 2–5 years: 0.25–2; 5–10 years: 0.41–0.89; > 10 years: 0.22–0.68 ng/ug crea) and mean urinary DS 21.22 ng/ug crea (SD 15.38; 4.33–45.8; NV: Age 0–2 years: 0.14–0.90; 2–5 years: 0.12–0.36; 5–10 years: 0.14–0.27; > 10 years: 0.03–0.22 ng/ug crea). Urine GAG concentrations were determined only at baseline because of financial limitations.

In all, the biochemical profile did not show any correlation between level of plasma I2S activity with clinical severity (Attenuated: mean of 0.37 nmol/mg plasma/4 h versus Severe: mean of 0.58 nmol/mg plasma/4 h). The specific types of GAGs were analyzed in only 10 patients with the severe phenotype, therefore GAG excretion could not be correlated with the clinical severity (Tables 7 and 8).

**Table 7:** Summary of plasma I2S activity and urinary GAGs concentration at baseline according to phenotype and age at diagnosis

	Plasma enzyme assay level (nmol/mg plasma/4 h)	Plasma enzyme assay level (uM/hr)	Urinary GAG concentration (mg/mg creatinine)	HS ng/ug Crea	DS ng/ug Crea
According to phenotype					
Attenuated (n = 8) M (SD) range	0.37 (0.31) 0.01–0.91	NA	465.73 (211) 145.35–692.59	NA	NA
Severe (n = 32) M (SD) range	0.58 (0.62) 0.01–2.34	0.124 (0.031) 0.09–0.17	547.04 (210.98) 126.1–911.46	35.87 (17.15) 14.85–58.4	21.22 (15.38) 4.33–45.8

	Plasma enzyme assay level (nmol/mg plasma/4 h)	Plasma enzyme assay level (> 4.45 uM/hr)	Urinary GAG concentration (mg/mg creatinine)	HS ng/ug Crea	DS ng/ug Crea
According to age at diagnosis					
< 6 years (n = 15)	0.50 (0.59) 0.01–1.96	0.1 (0.01) 0.09–0.11	548.72 (232.12) 238.09–911.45	52.12 (6.92) 42.28–58.4	17.51 (11.74) 4.33–31.67
6–10 years (n = 20)	0.55 (0.59) 0.01–2.34	0.11 N/A	494.72 (205.92) 126.1–778.62	20.48 (0.45) 20.16–20.8	26.8 (26.87) 7.8–45.8
> 10 years (n = 5)	0.39 (0.52) 0.04–1.15	NA	549.65 NA	21.19 (8.96) 14.85–27.53	18.12 (19.30) 4.47–31.76

**Table 8:** Summary of clinical and baseline biochemical characteristics

Pt #	Age at diagnosis (years)	Phenotype	Plasma enzyme (nmol/mg plasma/4 h)	Plasma enzyme (uM/hr)	Urine GAG (mg/g Crea)	Urine HS (ng/ug Crea)	Urine DS (ng/ug Crea)	On ERT	Outcome
1	1.8	Severe	0.01		891.6			Yes	Alive
2	3.75	Severe	0.17					Yes	Alive
3	9	Attenuated	0.75		363.3			Yes	Alive
4	8	Attenuated	0.31		629.73			Yes	Alive
5	7	Attenuated	0.91		302.04			Yes	Alive
6	5	Attenuated	0.01		443.76			Yes	Alive
7	6	Severe	0.12		567.6			Yes	Alive
8	7	Severe	0.07		690.65			Yes	Alive
9	15	Severe	0.04			27.53	4.47	No	Alive
10	10	Attenuated	0.31		145.35			No	Alive
11	0.58	Severe	0.26		568.56			No	Alive
12	10	Severe		0.14		44.27	41.98	No	Alive
13	5	Severe	0.14		759.98			No	Alive
14	7	Attenuated	0.1		683.36			No	Alive
15	4	Severe	0.77		376.06			No	Alive
16	7	Severe		0.17		22.64	10.36	No	Alive
17	4	Severe	0.07			42.28	4.33	No	Alive
18	2	Severe	0.09			58.4	12.6	No	Alive
19	23	Attenuated	0.34					No	Alive
20	13	Severe	0.04			14.85	31.76	No	Alive
21	9	Severe	0.06			20.8	45.8	No	Alive
22	2.5	Severe		0.11		54.66	31.67	No	Alive
23	3	Severe		0.09		53.13	21.42	No	Alive
24	4	Severe	1.96		339.87			No	Alive
25	7	Severe		0.11		20.16	7.8	No	Alive
26	5	Severe			472.73			No	Alive
27	9	Severe	1.08		626.57			No	Alive
28	7	Attenuated	0.26		692.59			No	Deceased
29	3	Severe	0.93		485.0509			No	Deceased
30	7	Severe	0.53					No	Deceased
31	7	Severe	0.742		778.6195			No	Deceased
32	5	Severe	1.1		238.0952			No	Deceased
33	6	Severe	1.08		126.1			No	Deceased
34	7	Severe	0.1		284.49			No	Deceased
35	14	Severe	0.94		743.35			No	Deceased
36	8	Severe	0.63		477.76			No	Deceased
37	4	Severe	0.525		911.4583			No	Deceased
38	6	Severe	2.34		535.221			No	Deceased
39	12	Severe	1.15		549.65			No	Deceased
40	6	Severe	0.01		517.45			No	Deceased

#### Mortality

At the time of review, 13 of the 40 (32.5%) have died, all coming from the untreated cohort. Majority (12/13; 92.3%) of the deceased patients were diagnosed with moderate to severe cognitive impairment, with only 1 patient diagnosed with mild ID. The mean age at death was 15.22 years old (SD 2.72; range 11.75–19). Pulmonary or cardiac causes were the only causes of death, with 11/13 (84.62%) deaths attributed to complications from pneumonia and 2/13 (15.38%) deaths due to progression of underlying cardiac disease.

#### Safety profile

Initially, there were 9 patients who started ERT in the Philippines. Of the 9, 4 had infusion-related reactions (IRRs), manifesting as anaphylaxis in 1 patient (11.11%) and urticarial rash in 3/9 (33.33%) patients. There were no non-IRRs recorded in the treatment group.

## Discussion

The first manuscript that described the clinical, biochemical and molecular characteristics of 23 Filipino patients with Hunter syndrome was published in 2017 [13]. Since then, around 36 more patients were diagnosed and listed in our local LSD registry, and ERT with IDS was started in 9 patients. This is a follow-up study, describing the clinical outcomes in two subsets of MPS II patients—idursulfase-treated and untreated. Although there are inherent limitations in this study—including a lack of more objective and standardized diagnostic assessments, variation in number of patients from baseline to each follow up period and the limited number of patients that precludes statistically significant comparison—it still provides valuable insight into the effects of therapy among our Filipino cohort. In general, ERT seems effective in reducing GAG storage as evidenced by resolution of hepatosplenomegaly, and results to a general trend of improvement in clinical endpoints such as growth, respiratory involvement, cardiac disease, joint ROM, and mortality rate.

This more recent data shows that the mean age at diagnosis remains at 7 years old, with delay of almost 5 years from symptom onset to biochemical confirmation. Compared to data from HOS [6] and the UK study, [5] where the mean ages at diagnosis were 1.5 and 1.2 years, respectively, there is significant delay among Filipino patients. The same reasons cited by Chiong et al., such as late recognition of this multi-system disorder and inadequate health system infrastructure and referral system, still hold true [13]. The early clinical manifestations among our cohort are consistent with previous reports, which were observed by the caregivers at an average of 2 years old. The data suggest that early recognition by caregivers need to be promoted, alongside education of primary care physicians on rare diseases.

All patients in this review were biochemically-confirmed. The level of plasma I2S activity was not predictive of clinical severity, which is in keeping with previous findings [13].

Management guidelines for the treatment of Filipinos with MPS II recommend ERT for any patient with a documented biochemical diagnosis, without severe cognitive impairment, and with at least one clinical manifestation that is still deemed treatable and not too far advanced to be addressed by ERT [14]. However, due to the prohibitive cost of enzyme plus the lack of government funding for orphan drugs, only 5 patients could qualify to receive ERT via Sanofi Genzyme’s International Charitable Access Program (ICAP). Two more siblings were able to start ERT through support from the Department of Health. However, the funding could only sustain treatment for 10 months. Two more patients were started on IDS under a multi-center experimental study. They were both assessed with severe cognitive impairment and were started on therapy at a mean age of 4.7 years. Overall, the mean age at start of treatment for our cohort was 14 years old, which is considerably later than their counterparts according to HOS, whose age at first treatment was 6.2 years old [6].

Short stature is a well-known feature of MPS II and is most evident after 8 years old [15]. Prior to this, height was observed to remain within normal range and even showed faster growth rates [15, 16]. However, this distinctive growth pattern was not apparent in our Filipino cohort, wherein 32% of patients 8 years and below were already found to be stunted. This may be due to ethnic differences and could be evaluated further by correlating with their respective mid-parental heights. As the patients age, height seem to slow down more considerably compared to unaffected peers as evidenced by lower mean increase in height and more percentage of stunted patients in the older than in younger age group. This also suggests that Filipino patients are unable to mount an adequate pubertal growth spurt, as expected, although growth velocity will give a more precise information. Consistent with published reports [12, 15], ERT has a positive effect on linear growth since treated patients had a greater increase in height than untreated age-matched patients.

Natural history data shows that MPS II patients tend to be heavier than their unaffected peers until 9 years old, and their BMI remain high until 14–16 years [16]. But this pattern was also not observed among our cohort since only 18% of patients in that age group were recorded to be overweight (WHO weight-for-age z-score above + 2), although body mass index would be a better indicator. Data on the effect of ERT on weight is more limited, and has only been studied by Tomanin et al. [17]. Similar to their analysis, our data suggests a negative trend for weight among untreated patients as there is greater percentage of underweight patients recorded in the older, untreated age group. Since the treated cohort demonstrated greater total weight gain compared with untreated group, it further supports mitigation of the negative effect on overall growth by ERT. Moreover, some untreated patients were found to have regression in growth (ie. shorter or lighter) at the end of the study period. These may be attributed to progression of joint contractures leading to decreases in height measurements, poor nutrition due to progression of somatic symptoms leading to emaciation, [18] or simply interobserver variability. Several factors, such as age at start of ERT, type of mutation, and cognitive impairment, were found to impact growth parameters, [12, 15] but their correlation remains to be explored among our Filipino Hunter syndrome patients.

The neurocognitive phenotype of MPS II is more heterogenous than previously thought [5] and needs further research among our patients. But according to the traditional binary classification, 80% of our patients fall within the early progressive or severe type, which is higher that the reported 61.5% from HOS [6].

Respiratory compromise is present in majority of our patients, as expected, most commonly presenting as OSA. The progression of airway obstruction seem more relentless in the untreated group as there is a higher absolute number of patients needing positive pressure assistance and tracheostomy coming from this cohort. Since FVC, the more objective parameter to assess pulmonary function in MPS II patients, [4] is not part of our routine monitoring, we cannot definitely determine whether ERT has any impact on respiratory function. Needless to say, this is worth looking into because improved pulmonary function would in turn lead to better growth, endurance, and survival [4].

Our findings on the positive effects of ERT on cardiac function are in keeping with published reports, [5, 6, 8] notably improvement in left ventricular dimensions. With regards to its effect on valvular disease, more data from our cohort is needed. As expected of the natural course of MPS II, there was a progression of cardiac disease when left untreated, evident in about half of our untreated cohort after 2 years.

Hepatosplenomegaly based on palpation was evident among our cohort, with liver more affected than spleen. Consistent with reports from HOS [6] and Japan Elaprase Treatment (JET) study, [8] ERT is effective in reducing the liver and spleen sizes, with continued improvement observed at year 1 and year 2 of follow up. This data suggests that ERT is indeed effective in reducing GAG storage, however routine urinary GAG measurement and abdominal magnetic resonance imaging or ultrasound will provide a more accurate information [6].

We have limited data to describe the effects of ERT on 6MWT. However, as expected, our untreated cohort have suboptimal results reflected by below average distance covered recorded during follow up. Data on joint mobility is hard to interpret because of heterogenous patient characteristics and measurements [6]. To date, two studies have shown evidence of joint mobility improvement with ERT—the JET study (albeit not statistically significant) [8] and the 53-week double-blinded study by Muenzer et al. (statistically significant improvement in shoulder ROM) [19]. While our data is limited and is confounded by interobserver variability, it suggests a positive impact of idursulfase treatment on joint mobility based on higher degree of improvement on more joints after 2 years of ERT. Moreover, worsening of ROM was observed on more joints in untreated cohort on follow up. Overall, our physical rehabilitation data underscores the need to consistently include 6MWT and joint ROM during follow up of MPS II patients. In particular, 6MWT is very informative because it is an integrated assessment of cardiac, respiratory, and musculoskeletal functions [4]. It also emphasizes that ERT should be coupled with consistent rehabilitation follow up and motivation of caregivers towards chronic illness, in order to have a more significant improvement in the functional outcomes of these patients.

Perhaps one of the most significant benefits of ERT is on patient survival, as documented by Burton et al. [9]. Although our sample size is mathematically small, it is still worth noting that all patients undergoing ERT are still alive at the time of review at a mean age of 16.14 years. Deceased patients succumbed on the second decade of life, most commonly as a result of pulmonary infection, which is the typical course for untreated patients [20–22]. A higher degree of cognitive impairment is associated with increased mortality, [9] which may explain why all but one of our deceased patients were categorized under the severe phenotype.

In our cohort, only IRRs were reported in majority, classified as mild in most cases with only one severe AE. The anaphylactic reaction was successfully managed outside of the ICU-setting. Recurrence of urticarial reactions was not observed, most likely due to premedication with antihistamine and/or corticosteroids and a 3-h infusion time. It has been suggested that IRR rates were higher in patients who were found to have developed antibodies, [23] therefore determination of antibody formation may provide more information on the safety and efficacy of ERT among our local cohort.

In conclusion, ERT is generally well-tolerated and effective in reducing GAG storage and improving clinical endpoints among our Filipino MPS II patients. Meanwhile, natural disease progression was observed in our untreated patients. As these treated patients are expected to receive long-term ERT, more objective studies, standardized assessments, as well as upkeep of the LSD registry should be undertaken in order to more accurately monitor the overall effect of therapy. It would also be interesting to evaluate the psychosocial impact of ERT in terms of amelioration of somatic symptoms juxtaposed against the burden of weekly injections. That being said, supportive measures remain to be standard of care for majority of our MPS II patients due to the prohibitive cost of idursulfase. Holistic well-being of our patients, therefore, will depend on continued supervision by a multidisciplinary team of experts, in partnership with committed caregivers and support from national health department.

## Data Availability

The datasets generated and/or analyzed during the current study are not publicly available because they are protected by the Data Privacy Act Data Privacy Act of 2012 (Republic Act 10173), but are available from the corresponding author on reasonable request.
